# Semaglutide: Double-edged Sword with Risks and Benefits

**DOI:** 10.26502/aimr.0189

**Published:** 2025-01-10

**Authors:** Lekha Pillarisetti, Devendra K. Agrawal

**Affiliations:** 1 Department of Translational Research, College of Osteopathic Medicine of the Pacific, Western University of Health Sciences, Pomona, California 91766 USA.

**Keywords:** Alzheimer’s Disease, Diabetes mellitus, Diabetic retinopathy, Drug interactions, Hyperglycemia, Insulin resistance, Medullary thyroid carcinoma, Pancreatitis, Pulmonary aspiration, Semaglutide, Stroke, Weight loss

## Abstract

Type 2 Diabetes Mellitus therapy has evolved over the years to now include a new class of therapeutics, semaglutide. This article reviews the mechanism of action and formulation of semaglutide therapy, potential benefits, contraindications, adverse effects, and drug interactions. Oral and subcutaneous semaglutide therapies have shown effectiveness in improving glycemic control, weight loss, and reducing cardiovascular risks associated with diabetes mellitus. Semaglutide has also shown potential in being used as a therapeutic strategy in Alzheimer’s disease due to its anti-neuroinflammatory effects and being used to treat polycystic ovary syndrome. However, semaglutide therapy is also associated with concerning adverse effects like acute pancreatitis, anesthetic risks like pulmonary aspiration or residual gastric content, acute kidney injury, acute gallbladder injury, nonarteritic anterior ischemic optic neuropathy and diabetic retinopathy. Contraindications of semaglutide include history of medullary thyroid carcinoma or multiple endocrine neoplasia syndrome type 2, and pregnancy. Drug interactions to consider with semaglutide therapy include those also used in diabetes treatment, like metformin, as well as anti-psychotics, due to anti-psychotics associated weight gain. The findings of this article emphasize the need for a cross-disciplinary approach to understand the molecular mechanisms and clinical implications of semaglutide on patients with complex medical histories and treatment regimens. The potential anesthetic risks of semaglutide therapy warrant careful consideratiion with ethical concerns. Further studies can assess if there is a need to modify pre-operative guidelines to account for patient using semaglutide and how delayed gastric emptying and constitpation will affect surgical outcomes and complications. While semaglutide therapy for diabetes mellitus has been established, there is a need for extensive research on repurposing semaglutide in neurodegenerative disease treatment.

## 1. Introduction

Islet cells within the pancreas contain two important classes of cells: insulin-producing beta cells and glucagon-producing alpha cells, which regulate blood glucose. In diabetes mellitus (DM), this balance is disrupted, leading to hyperglycemia, increased blood glucose concentrations [1, 2]. Type 1 DM results from autoimmune destruction of beta cells, causing insulin deficiency, thus an inability to uptake glucose from the blood. Type 2 DM (T2DM) develops gradually due to insulin resistance, in turn the body is less sensitive to insulin, thus decreasing glucose uptake from the blood [2].

The global prevalence of T2DM has exhibited a significant upward trajectory in recent years, with epidemiological data indicating a substantial increase in both incidence and prevalence rates. According to the Global Burden of Disease Study 2019, the age-standardized prevalence of T2DM reached 6.28% globally, translating to approximately 462 million affected individuals [1]. Projections suggest a continued escalation, with an estimated prevalence of 7,079 out of 100,000 individuals by 2030, underscoring the growing public health concern [1].

The pathophysiological effects of T2DM are multifaceted, encompassing issues in glucose homeostasis, cardiovascular function, and weight gain. Chronic hyperglycemia can result in microvascular and macrovascular complications, including peripheral neuropathy, nephropathy, ophthalmic abnormalities, and cardiovascular disease [3]. Cardiovascular morbidity and mortality are significantly elevated in T2DM patients, with a two-to-four-fold increased risk of adverse cardiovascular events compared to non-diabetic individuals [1, 3, 4]. Moreover, the intricate relationship between T2DM and obesity contributes to a state of chronic low-grade inflammation and insulin resistance, further exacerbating metabolic dysfunction [1, 5].

Semaglutide, a glucagon-like peptide-1 (GLP-1) receptor agonist, has emerged as a promising therapeutic for T2DM management [5]. Its pharmacological profile is characterized by high affinity and specificity for the GLP-1 receptor, coupled with a prolonged half-life of about one week due to albumin binding [5]. This research article focuses on understanding the mechanism and forms of semaglutide therapy, efficacy, contraindications, any adverse effects and common drug interactions.

### I. Mechanism of Action

In developing semaglutide, prioritizing the fatty acid moiety and linking chemistry was crucial for achieving high albumin affinity and GLP-1 receptor (GLP-1R) potency, enabling prolonged exposure of the GLP-1 analogue. As a result, semaglutide incorporates two amino acid substitutions (Aib8, Arg34) and is modified at lysine 26 [6]. This modification makes the drug's half-life of 165 hours [7].

At the molecular level, semaglutide's mechanism of action involves the GLP-1 receptor (GLP-1R), a G-protein coupled receptor [8]. Upon binding to GLP-1R, semaglutide leads to increased intracellular cyclic adenosine monophosphate (cAMP) levels, activating protein kinase A (PKA). PKA is involved in the synthesis and secretion of insulin but inhibits the release of glucagon. Increased levels of cAMP can also activate the Rap1 via EPAC (Exchange Protein directly Activated by cAMP), which is involved in regulation of insulin secretion [8, 9]. Semaglutide, mimicking GLP-1 signaling, can also activate the PI3K/Akt pathway involved in maintenance and viability of pancreatic β-cells [8]. In pancreatic β-cells, this signaling cascade also enhances glucose-dependent insulin secretion and sensitivity of peripheral tissues to insulin. Simultaneously, semaglutide suppresses glucagon secretion by pancreatic α-cells, contributing to improved glycemic control [8-10].

Semaglutide, like GLP-1, also has an effect on receptors in the peripheral and central nervous systems. In the context of metabolic disease, uncontrolled T2DM can lead to peripheral neuropathy. GLP-1 receptors are found diffusely in both the peripheral and central nervous systems. It has been suggested that semaglutide may exert benefits in improving symptoms like peripheral neuropathy, beyond glycemic control [9, 11]. The central effects of semaglutide on energy homeostasis are mediated through its action on GLP-1 receptors in the hypothalamus and brainstem, leading to reduced appetite and food intake [11, 12]. The mechanism of reduced appetite is complex but appears to involve neural circuits of the vagus nerve [5, 12]. Additionally, semaglutide has been shown to delay gastric emptying, further contributing to its weight loss effects [12].

### II. Formulations of Semaglutide

Semaglutide is available in two formulations: oral and subcutaneous. Oral semaglutide therapy may be preferential to subcutaneous depending on patient preference or in cases when metformin is inappropriate [9].

#### A. Oral Semaglutide Therapy

Oral semaglutide therapy should start at 3 mg once daily for a month before doses are increased to 7 mg once daily. If necessary, after a month of maintenance, dosage can be increased to 14 mg once daily [9, 13]. This formulation should be taken on an empty stomach, with a 30-minute wait before eating or taking other medications to optimize absorption, as food and increased fluid intake can affect bioavailability. The absorption enhancer sodium N-(8-[2-hydroxybenzoyl]amino) caprylate (SNAC) is used to improve its minimal bioavailability, which ranges from 0.4% to 1% [9, 13, 14]. Peak concentration is reached approximately 1-hour post-dose, with steady-state achieved after 4-5 weeks. An example of a brand name therapeutic on the market is Rybelsus [9, 13].

Recent clinical trials, like OASIS 1, have shown that overweight or obesity adults without T2DM should take oral semaglutide 50 mg once per day best weight loss results [15].

#### B. Subcutaneous Semaglutide Therapy

Subcutaneous semaglutide therapy should start at 0.25 mg once weekly and after 4 weeks, dosage can be increased to 0.5 mg once weekly. If necessary, dosage can be increased to 1 mg once weekly after another 4 weeks. This route offers a high bioavailability of 89%, with peak concentrations reached within 1-3 days and steady-state occurring after 4-5 weeks [9, 13]. The subcutaneous administration is flexible regarding meal timing, with injection sites rotated weekly among the abdomen, thigh, and upper arm [13]. Examples of brand name therapeutics on the market include Ozempic and Wegovy [5].

Both formulations share an elimination half-life of about one week, remaining in circulation for around five weeks post-last dose, with clearance rates of 0.05 L/h for subcutaneous and 0.04 L/h for oral in healthy individuals [9, 13]. Elimination occurs primarily through the urine and feces.

### IV. Efficacy in Clinical Trials

#### A. Cardioprotective Effect

The SUSTAIN-6 trial provided compelling evidence for the cardiovascular benefits of semaglutide, demonstrating a 26% reduction in the risk of major adverse cardiovascular events in T2DM patients with elevated cardiovascular risk [16].

Semaglutide, a GLP-1 receptor agonist, has demonstrated superior cardioprotection for people with T2DM compared to other treatments. Clinical trials like SUSTAIN and PIONEER found that it reduced major adverse cardiovascular events (MACE) by approximately 26%, outperforming other diabetes drugs (17). Its benefits extend beyond glycemic control, including weight reduction and anti-inflammatory effects, which collectively lower cardiovascular risks. These advantages make semaglutide especially effective for T2DM patients with pre-existing cardiovascular conditions [9, 13].

Additionally, patients with Heart Failure With Preserved Ejection Fraction (HFpEF) and obesity, semaglutide produced large improvements in HFpEF related symptoms [18]. HFpEF is when the ventricles of the heart are unable to fill or eject blood as they should. Thus, patients suffer from dyspnea, fatigue, congestion and heart failure (HF) [19]. Using mouse models, Withaar et al. came the same conclusion, finding that semaglutide treatment significantly reduced messenger RNA levels of cardiac stretch marker and inflammatory markers like interleukin-6, which is consistent with functional improvements. Semaglutide treatment is also found to decrease myocardial diastolic stiffness and improve actin-myosin and muscle contraction pathways [20]. There must be a pathway where semaglutide are able to exert anti-inflammatory effects, which should be the focus of more studies.

Semaglutide therapy also reduced incidence of any stroke when compared to a placebo through significant reductions in risk of small-vessel occlusion in a post hoc analysis of the SUSTAIN 6 and PIONEER 6 trials [21]. In addition, Avgerinos et al., conducted a meta-analysis and concluded that semaglutide therapy was associated with a 39% decrease In the risk of ischemic stroke [22]. Semaglutide therapy also seems to decrease incidence of stroke comparative to other therapies, specifically a dipeptidyl peptidase-4 inhibitor [23]. While there have been studies on the incidence of stroke in patients on semaglutide compared to a specific T2DM therapy, there is a need to understand how T2DM therapies generally compare to each other in reducing risk of stroke.

Maskery et al. conducted a systematic review confirming previous studies findings that semaglutide therapy is associated with decreased incidences of stroke. The study also concluded semaglutide therapy was associated with reduced infarct volume, apoptosis, oxidative stress and inflammation alongside increased neurogenesis, angiogenesis and cerebral blood flow [24].

#### B. Effects on Alzheimer’s Disease

Semaglutide therapy has also garnered attention for its potential therapeutic effects for Alzheimer’s Disease. Alzheimer’s Disease is a neurodegenerative disease characterized by dementia and cognitive impairment in language, comprehension, memory and more [25]. Specifically, semaglutide therapy has been associated with associated with decreased amyloid-beta plaque deposition and neuroinflammation. It’s effects on the central and peripheral nervous system are thought to mediate these effects, party through ability to cross the blood-brain barrier [26]. While the specific mechanism of action is unknown, it is thought that since neurons rely on consistent regulation of glucose transporters to prevent neurodegeneration, a pathway that semaglutide therapy directly modulate, it is able to exhibit pleiotropic effects [26]. Pooled data from three randomized controlled trails and a nationwide registry-based cohort have consistent findings. Nørgaard et al. found that rates of dementia were lower in patients undergoing G1PR agonists compared to a placebo in both the randomized control trail patient data and nationwide cohort data [27]. There is a need for more studies to explore the idea of repurposing semaglutide therapy for Alzheimer’s Disease treatment.

#### C. Glycemic Control

One of the main goals of semaglutide therapy is a reduction in HbA1c levels to improve glycemic control. Subcutaneous semaglutide, administered once weekly, has shown a 1.5–1.8% reduction in HbA1c levels over 30–56 weeks in various SUSTAIN trials. For example, in SUSTAIN 1, subcutaneous semaglutide reduced HbA1c by 1.6% after 30 weeks, making it superior to several comparators, including sitagliptin, liraglutide, and insulin glargine [28].

In contrast, oral semaglutide, taken once daily, demonstrated HbA1c reductions ranging from 1.0–1.4% in the PIONEER trials, achieving a 1.4% reduction after 26 weeks in PIONEER 1 [28]. Although effective, this was slightly less potent compared to subcutaneous semaglutide. Nevertheless, oral semaglutide achieved similar efficacy to liraglutide and better outcomes than sitagliptin or empagliflozin. A PIONEER REAL pooled analysis also indicated that individuals on oral semaglutide treatment experience better glycemic control and weight loss across various age groups and durations of T2DM [29].

The differences in glycemic control between the two formulations may be influenced by the consistency of plasma concentration, with subcutaneous administration yielding a more stable pharmacokinetic profile.

Recent studies have also looked at new mechanisms of action utilizing semaglutide. Schneider et al. 2024 looked into possible mechanisms for a long-acting version of the semaglutide. Semaglutide was attached to hydrogel microspheres with a cleavable linker, designed for release over about one month. In mice, a single subcutaneous dose showed a release half-life of approximately 36 days, leading to a 20% weight loss over one month, similar to twice daily semaglutide doses [30]. Simulations suggested that this microsphere formulation could allow for once-monthly human dosing while maintaining effective drug levels, potentially reducing adverse side effects.

#### D. Weight Loss

In SUSTAIN 1 and PIONEER 1 trials, with patients managing early-stage diabetes with diet and exercise alone, subcutaneous semaglutide 1.0 mg led to a mean weight reduction of 4.5 kg, whereas oral semaglutide 14 mg achieved a 3.7 kg reduction [28]. These effects were superior to those observed with placebo and other active comparators.

In the SUSTAIN 2, PIONEER 3, and PIONEER 7 trials involving patients with established Type 2 Diabetes (T2D) on one or two oral antidiabetic drugs (OADs), both subcutaneous semaglutide (0.5 mg and 1.0 mg) and oral semaglutide (7 mg, 14 mg, and flexibly dosed) resulted in significantly greater body weight reductions compared to sitagliptin. Specifically, subcutaneous semaglutide 1.0 mg led to a greater weight loss than other GLP-1 receptor agonists: exenatide ER 2.0 mg, dulaglutide 1.5 mg , and liraglutide 1.2 mg [28].

Additionally, oral semaglutide 14 mg showed a significant reduction in body weight compared to liraglutide 1.8 mg in the PIONEER 4 trial [28].

#### E. Chronic Kidney Disease (CKD)

The FLOW clinical trial investigated whether semaglutide can slow the progression of CKD in people with T2DM. Participants were randomly assigned to receive either subcutaneous semaglutide or a placebo, administered weekly. Among 3533 randomized participants, the semaglutide group had demonstrated benefits for kidney-specific outcomes, cardiovascular death, and other secondary outcomes, including a slower decline in kidney function [31].

#### F. Polycystic Ovary Syndrome (PCOS)

PCOS is a major cause of infertility in women of reproductive age, with oral contraceptives being commonly used for treatment. However, these drugs only target a few symptoms and may have severe adverse effects. Repurposing existing drugs could be a feasible option to improve PCOS outcomes with less adverse events [32]. The use of GLP-1R agonists in women with PCOS could provide weight loss effects, as well as improve hyperinsulinism, hyperandrogenism, normalize total testosterone, insulin resistance markers, and total cholesterol. A total of 176 participants from four RCTs were included in a systematic review study, where GLP1-R agonist use was associated with a significant reduction in waist circumference, BMI, serum triglycerides, and total testosterone levels compared to placebo. However, no significant differences were found in total cholesterol and insulin resistance marker levels. Among 112 patients with reported adverse events, 49 experienced mild side effects such as nausea and abdominal pain [33].

Another study also showed that semaglutide effectively reduces body weight in obese PCOS patients who did not respond to lifestyle changes. After three months of treatment, most patients lost weight, with 80% achieving a weight loss of at least 5%, and improvements in insulin resistance and fasting blood glucose were observed, with minimal side effects. Those who continued treatment for six months experienced further weight loss and normalization of menstrual cycles [34].

### V. Contraindications

#### A. Medullary Thyroid Carcinoma and Multiple Endocrine Neoplasia Syndrome (MEN2)

Multiple Endocrine Neoplasia Syndrome (MEN2) is a rare, genetic disorder that affects the endocrine glands and can cause tumors in the thyroid gland, parathyroid glands, and adrenal glands [35, 36]. It is characterized by development of medullary thyroid carcinoma (MTC). MTC is a type of thyroid cancer that arises from parafollicular C cells [37].

In a systematic review encompassing 10 randomized controlled trials (RCT), isolated instances of papillary and MTC were reported, each accounting for less than 1% of the study populations. This would indicate that there is no substantial risk of thyroid cancer linked to semaglutide use when evaluating the large sample sizes [38].

However, a study by Bezin et al. revealed that the GLP-1 receptor agonists use, like semaglutide, for 1 to 3 years was associated with an increased risk of all types of thyroid cancer, with an adjusted hazard ratio of 1.58 and an adjusted hazard ratio of 1.78 specifically for MTC [39]. These results contrast with earlier studies indicating no significant thyroid cancer risk associated with semaglutide. The discrepancies in findings may be attributed to differences in study design, the populations studied, and potentially the formulations of GLP-1 receptor agonists examined. Due to these findings, semaglutide is contraindicated in patients with a personal or family history of MTC or MEN2 syndrome [5].

#### B. Pregnancy

Current clinical trial data is insufficient to define an association between complications in pregnancy and semaglutide therapies, but there are instances of unfavorable fetal outcomes when using G1PR agonists [40]. There have also been sporadic events of complications. In one such case, a 40-year-old female exposed to semaglutide Ozempic had a child with an atrial septal defect that resolved spontaneously 3 years after birth [41]. But due to these sporadic cases, the manufacturer advised stopping this semaglutide treatment 2 months prior to conception of the fetus [42].

In a multinational population-based cohort study that included 51,826 pregnant women diagnosed with T2DM and their infants, the standardized prevalence of major congenital malformations was found to be 8.3% among infants exposed to GLP-1 receptor agonists (GLP1-RA) during the periconceptional period [43]. When comparing GLP1-RA to insulin, there was no significant increase in the risk of major congenital malformations, with an adjusted relative risk of 0.95 for infants exposed to GLP1-RA.

Product labeling indicates that animal studies have suggested a potential for reproductive toxicity at maternally toxic doses for semaglutide, dulaglutide, exenatide, and liraglutide. Additionally, an increased risk of malformations was noted for liraglutide and semaglutide at doses similar to those used in human treatments [43].

### VI. Adverse Effects (AE)

#### A. Common AEs

##### 1) Gastrointestinal Effects

The most common symptoms associated with the use of GLP-1 receptor agonists are gastrointestinal symptoms, mainly nausea, vomiting, diarrhea, constipation, and abdominal cramps [44].

Studies suggest that continued treatment with semaglutide therapy may diminish over time as patients adjust. Wharton et al. 2022 found in the STEP 4 trial, in the randomized 803 participants GI AEs with onset during the randomized period were reported in 41.9% of participants receiving continued semaglutide 2.4 mg treatment, compared with 26.1% of participants that switched to placebo [45]. In participants receiving continued semaglutide 2.4 mg treatment, the prevalence of nausea, diarrhea and constipation decreased over time, and the prevalence of vomiting remained low [46].

##### 2) Residual Gastric Content (RGC)

The mechanism of semaglutide therapy includes the effect of delaying gastric emptying. This can result is AEs like residual gastric content (RGC). In a retrospective cohort study by Korlipara et al., after accounting for confounding factors, semaglutide use was associated with a 6% increase of retained solid gastric contents [47]. Another retrospective chart review found a significant association between semaglutide therapy and RGC in patients that underwent elective esophagogastroduodenoscopy [48].

Semaglutide use is also associated with gastroparesis, the state of delayed stomach emptying, which can cause abdominal discomfort, nausea, bloating and postprandial fullness [49]. In this case study by Chaudhry et al. discussed as case report of a patient who suffered from these symptoms, compounded by semisolid food retained in the fundus of the stomach. Residual gastric content was highly consistent with the patient’s diagnosis of gastroparesis. Symptoms resolved after discontinuing semaglutide use [49]. There is a need to understand the role of semaglutide in inducing gastroparesis and whether there is a consistent association between semaglutide therapy and gastroparesis.

Common AEs of semaglutide discussed can be managed by using the three E method: Education, Escalation, and Effective management. First, educate patients about potential side effects, reassuring them that mild symptoms often resolve over time. Next, escalate the dosage gradually to minimize side effects, adjusting the pace based on each individual’s tolerance. Lastly, implement effective management strategies, including eating small meals more frequently, hydration, and, if necessary, pharmacological treatments such as short term use of antiemetics [50].

#### B. Severe AEs

##### 1) Gastrointestinal Effects

A study examined adverse effects (AE) associated with semaglutide therapy using a FDA adverse event reporting system. Shu et al. 2022 found severe gastrointestinal adverse events (AEs) related to semaglutide treatment were reported in 1,778 GI cases and 3,601 overall cases, resulting in 40 (2.25%) and 102 (2.83%) deaths, respectively [51]. The most common severe outcomes included hospitalizations, with 1,103 (62.04%) from GI AEs and 2,311 (64.18%) from overall AEs. Of the GI reports, 40.25% came from healthcare professionals (n = 2,183), while 59.75% were from consumers (n = 3,240) [51].

##### 2) Nonarteritic Anterior Ischemic Optic Neuropathy (NAION)

A retrospective matched cohort study assessed the association between semaglutide and nonarteritic anterior ischemic optic neuropathy (NAION), which is the leading cause of blindness among adults, among 16,827 patients from December 2017 to November 2023. Among patients with T2DM, there was 8.9% incidence of NAION events in the semaglutide cohort and 1.8% in the non–GLP-1R agonist cohort. The hazard ratio (HR) for NAION was 4.28 for those prescribed semaglutide. In the overweight/obese cohort, there was a 6.7% incidence of NAION events in the semaglutide group and 0.8% % in the non–GLP-1R agonist cohort. The HR for NAION in this group was 7.64 [52].

These findings suggest an association between semaglutide and increased risk of NAION. However, as this was an observational study, further research is needed to establish causality.

There is no consistent, effective therapy for NAION other than discontinuation of semaglutide. Most possible therapies show mixed results or cannot provide long term efficacy (e.g. anti-VEGF, erythropoietin, hyperbaric oxygen, optic nerve sheath fenestration (ONSF)). Currently, none of these therapies are recommended, emphasizing the need for further research [53].

##### 3) Diabetic Retinopathy (DR)

Diabetic Retinopathy (DR) is a microvascular complication of diabetes mellitus due to damage to the retina. DR is one of the most frequent complications of DM and the leading cause of blindness in the adult population [54]. Studies are not conclusive as to whether DR is associated with semaglutide therapy or due to other risk factors [55].

In the SUSTAIN 6 trial, semaglutide therapy was associated with a significantly higher rate of diabetic retinopathy complication when compared to the placebo group [45]. Husain et al. 2019 in a randomized, double blind placebo controlled trial found the percentage of patients with adverse events related to diabetic retinopathy during the trial was 7.1% (113 of 1591 patients) with oral semaglutide and 6.3% (101 of 1592) with a placebo [56]. However, this was not significant and no association was found. In a retrospective 3 year study, Stevens et al. 2024 found semaglutide use was not associated with increased risk of progression of DR [57]. Currently, a long term clinical trial is investigating the effects of semaglutide on the development and progression of DR and is planned to conclude in 2027 (ClinicalTrials.gov number, NCT03811561).

Risk factors associated with the development of DR while using semaglutide include age and duration of living with T2DM. Individuals aged 60 or older or those having T2DM for 10 years or longer may be at higher risk [45]. In general, not accounting for therapy type, DR is more prevalent in Type 1 DM individuals, those with increased duration of living with DM, and higher levels of values for HbA1c, blood pressure, and cholesterol [58].

Management of DR for patients on semaglutide therapy include prophylactic treatment with vascular endothelial growth factor antagonism (anti-VEGF). Ranibizumab and aflibercept therapy in eyes can also reduce progression of DR providing a safe management option for patients on semaglutide who may experience rapid glucose corrections [59].

##### 4) Acute Pancreatitis

Pancreatitis is inflammation of the pancreas, and the pathophysiological cause can be issues with enzyme secretion [60]. Most case reports show that pancreatitis is an acute complication of semaglutide use and Masson et al. found no association of increased risk of acute pancreatitis between semaglutide therapy and placebo [61]. However, a recent case study reported by Dagher et al., shows semaglutide induced pancreatitis after four years of semaglutide therapy resulting in subsequent distributive shock and death in the patient [62]. Although this is a unique case, there is a need to understand if there is an association of four year semaglutide use with life threatening pancreatic complications, compounding risk factors and the mechanism by which it could occur.

Management of acute pancreatitis include prompt discontinuation of semaglutide along with the therapeutic interentions based on severity including fluids, pain medication, nutritional support, and antibiotic use [63, 64].

##### 5) Pulmonary Aspiration

Pulmonary Aspiration is one of the most high risk airway management complications. There have been cases of pulmonary aspiration in patients undergoing surgery due to delayed gastric emptying resulting in resident gastric content. Avraham et al. reports 2 cases of peri-operative pulmonary aspiration in patients undergoing semaglutide therapy. They report how a patient taking semaglutide may have RGC despite complying to pre-operative fasting rules [65]. Klein et al. reports a similar case where RGC had to be removed via bronchoscopy from the trachea and bronchi [66].

Fezza et al. defines the need for screening of patient semaglutide use prior to any major surgery to assess anesthetic risk of pulmonary aspiration [67]. There also is a need for studies focused on the prevalence of pulmonary aspiration and risk factors associated with patients undergoing semaglutide therapy.

To manage this complication, preventative measures like rapid sequence induction and tracheal intubation can be used. In pre-operation, gastric ultrasound can identify patients with a high risk of aspiration [65].

##### 6) Acute Kidney Injury

As mentioned, the FLOW clinical trial did demonstrate renoprotective benefits for patients with CKD on semaglutide [31]. However, caution is advised for patients with stage 3b-4 CKD and kidney function should be monitored, especially in those with severe gastrointestinal side effects that may lead to dehydration and increase the risk of acute kidney injury [68].

Case reports have also revealed a need to understand underlying complicating factors contributing to development of acute kidney injury on semaglutide therapy. A 49-year-old female with morbid obesity and controlled hypertension presented with 8 weeks of bilateral pedal edema, weight gain, and worsening kidney function after starting semaglutide 0.5 mg weekly for weight loss. Kidney biopsy revealed focal segmental glomerulosclerosis and acute interstitial nephritis. Given the possible association with the timing of semaglutide initiation, the medication was discontinued, leading to improvement in kidney function [69].

In another case study, a 68-year-old female with stage 3a CKD, diabetes, and other comorbidities experienced a significant rise in serum creatinine after starting semaglutide 0.25 mg weekly, along with nausea, vomiting, and proteinuria. [69].

Case studies, therefore, suggest that both volume depletion due to gastrointestinal side effects and other intrinsic mechanisms may contribute to renal dysfunction.

Acute kidney injury can be managed by discontinuing the semaglutide and a combination of steroid treatment and dialysis [69].

##### 7) Acute Gallbladder Injury

The labeling for some GLP-1R agonists approved for T2DM includes warnings about acute gallbladder disease, but some do not. Woronow et al. 2022 reviewed the FDA Adverse Event Reporting System for cases of acute cholecystitis (AC) associated with GLP-1R agonists lacking these warnings, identifying 36 cases [70].

Most cases were severe, with 30 patients requiring cholecystectomy, 2 resolving with medication and drug discontinuation, and 1 resulting in death. The median age was 55 years, and over half of the patients were female, with recent weight loss reported in nine cases. Time to AC onset varied, occurring within 90 days in nearly half the cases, and was shorter for patients on starting doses compared to those on maximum doses. Severe outcomes included pancreatitis and fatal liver necrosis, though confounding factors were present, like fatty liver disease and possible thalassemia minor [70].

In a meta-analysis of 76 RCTs involving 103,371 patients found that GLP-1 RA use was associated with increased risks of gallbladder or biliary diseases, including cholelithiasis, cholecystitis, and biliary disease. These risks increased at higher doses, for longer durations emphasizing a possible dose and duration dependent relationship [71].

Thus, there are cases linking GLP-1 RAs to AC include weight loss, suppression of cholecystokinin secretion, and reduced gallbladder emptying.

In a study reviewing the FDA Adverse Event Reporting System, primary treatment for acute gallbladder injury was a cholecystectomy, performed in 30 out of 36 cases. Two cases were resolved using ursodeoxycholic acid combined with discontinuation of GLP-1 receptor agonists [70].

Generally, semaglutide is a well-received therapy with minimal severe adverse events. However, future studies should follow patients longitudinally to see how these effects persist long term.

### VII. Drug Interactions

Patients diagnosed with T2DM are able to be treated with a host of therapies, mostly pharmacological [72]. Pharmacological drug interactions may impair efficacy of drugs or cause adverse drug reactions that can be life-threatening.

#### A. Metformin

Metformin is a diabetic treatment for T2DM. It is used to manage gestational diabetes, weight gain, prevent T2DM and treat polycystic ovarian syndrome [73]. In a study assessing concurrent drug administration and semaglutide treatment, metformin absorption was unchanged when administered concurrently with semaglutide. Delayed gastric emptying due to semaglutide somewhat prolonged absorption. Hausner et al 2017. noted no safety or tolerability issues when metformin and semaglutide were administered together [74]. This finding is supported by Bækdal et al. 2019 that found oral semaglutide did not significantly increase metformin exposure [75].

#### B. T2DM Drugs

Yang et al. 2024 found exposure of semaglutide appeared to be slightly non-significantly increased when oral semaglutide was administered with omeprazole versus oral semaglutide alone [76]. When co-administered with warfarin, digoxin, atorvastatin, ethinyl estradiol or levonorgestrel, there was no impairment in delivery or absorption [76]. However, due to the effect on gastric emptying there could be minimal effects with absorption of T2DM drugs when administered with semaglutide.

#### C. Anti-Psychotics

Ziprasidone, marketed as Geodon, is an antipsychotic that acts on dopamine D2 and serotonin 5-HT2A receptors and serves as a 5-HT1A receptor agonist. These actions help manage schizophrenia symptoms with minimal metabolic side effects. However, one of Ozempic’s mechanisms, slowing gastric emptying, may impact Geodon’s absorption, as demonstrated by in a case study where a patient’s supratherapeutic Geodon level was 238.7 ng/mL, while the recommended level is 220 ng/mL [77]. Thus, it is vital to consider how semaglutide can interact with antipsychotics to alter or prolong systemic effects.

There has been some concern that this drug interaction could interfere with the mechanism of antipsychotics leaving patients vulnerable to suicide ideation (SI) or depression. Wang et al. conducted a retrospective cohort study and found that semaglutide use was not significantly correlated to SI in patients with T2DM [78]. Another study by Wadden et al. considered once weekly 2.4mg semaglutide therapy and SI in post hoc analysis of STEP 1, 2, 3 and 5 clinical trials. They found no significant difference for depression or SI between semaglutide therapy and the placebo [79].

Additionally, semaglutide shows promise in treating antipsychotic-associated weight gain (AAWG). Evidence suggests it can effectively reduce weight and improve metabolic parameters in such cases, with its weekly dosing and tolerable side effects making it a good option, particularly for patients unresponsive to metformin [80].

### VIII. Conclusion

In conclusion, semaglutide represents a significant advancement in the pharmacological management of T2DM, offering improvements in glycemic control, cardiovascular outcomes, and obesity. Its multifaceted mechanism of action, targeting both central and peripheral pathways involved in glucose and energy homeostasis, positions it as a promising agent in the evolving landscape of metabolic and neurodegenerative disease management. Ongoing research continues to show its potential applications in non-diabetic indications and long-term safety profile, while pharmacological analyses will be crucial to assess efficacy and ensure patient safety.

## Figures and Tables

**Figure 1:**
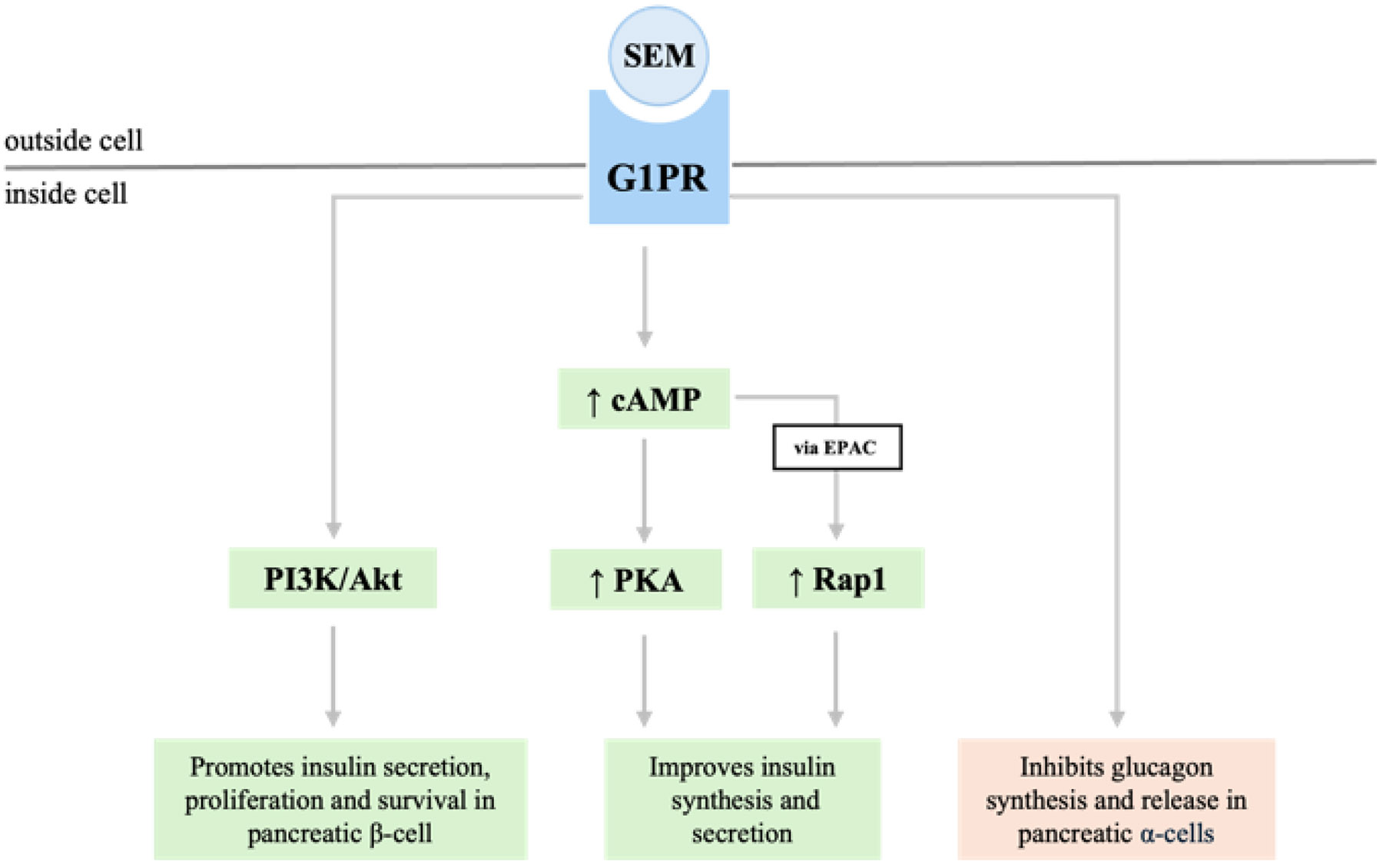
This flowchart depicts the downstream signaling pathways activated by semaglutide (SEM) binding to the GLP-1 receptor (GLP-1R). Key pathways include PI3K/Akt, which promotes insulin secretion, β-cell proliferation, and survival, and cAMP signaling via protein kinase A (PKA) and Exchange Protein directly Activated by cAMP (EPAC), enhancing insulin synthesis and secretion. Additionally, GLP-1R activation inhibits glucagon synthesis and release in pancreatic α-cells, improving glycemic control.

**Figure 2:**
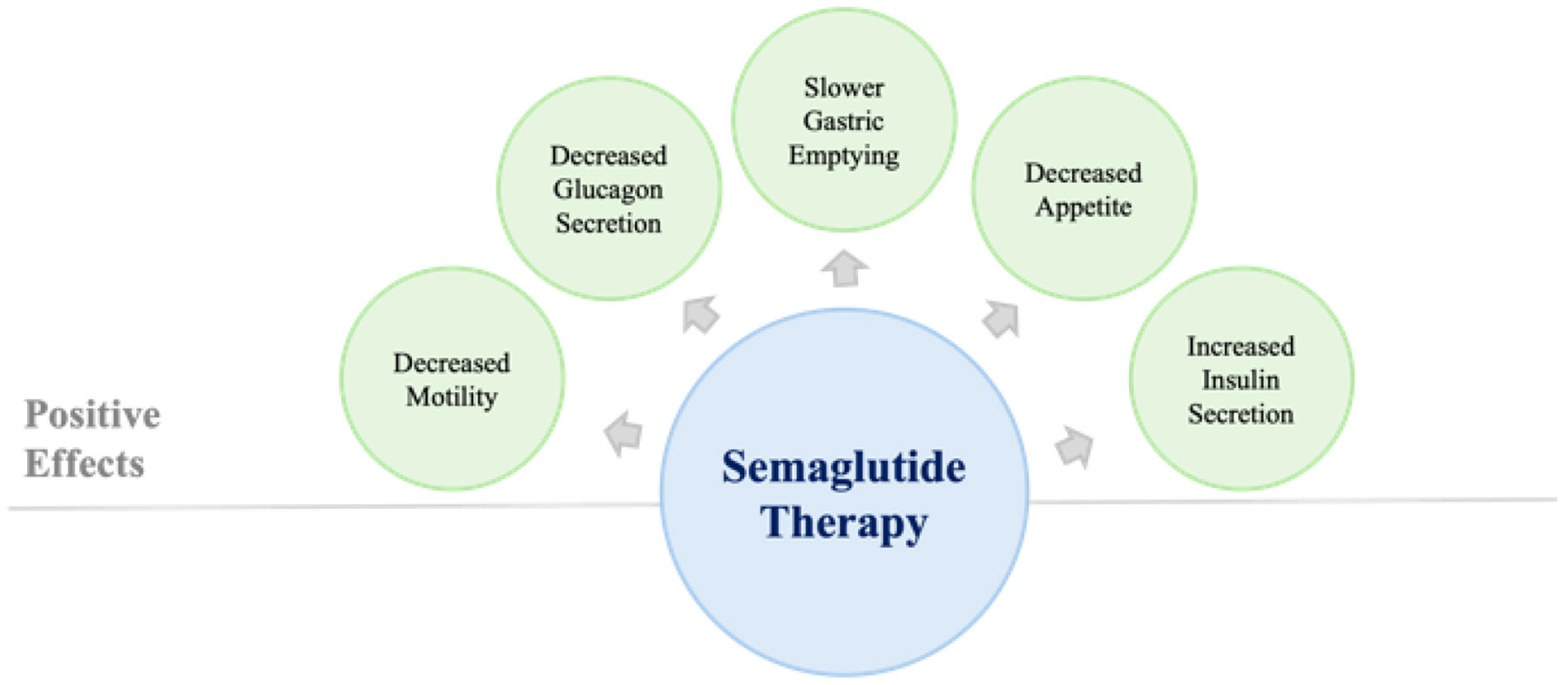
The figure depicts the positive effects of semaglutide therapies for patients with T2DM discussed in the mechanism of action.

**Figure 3:**
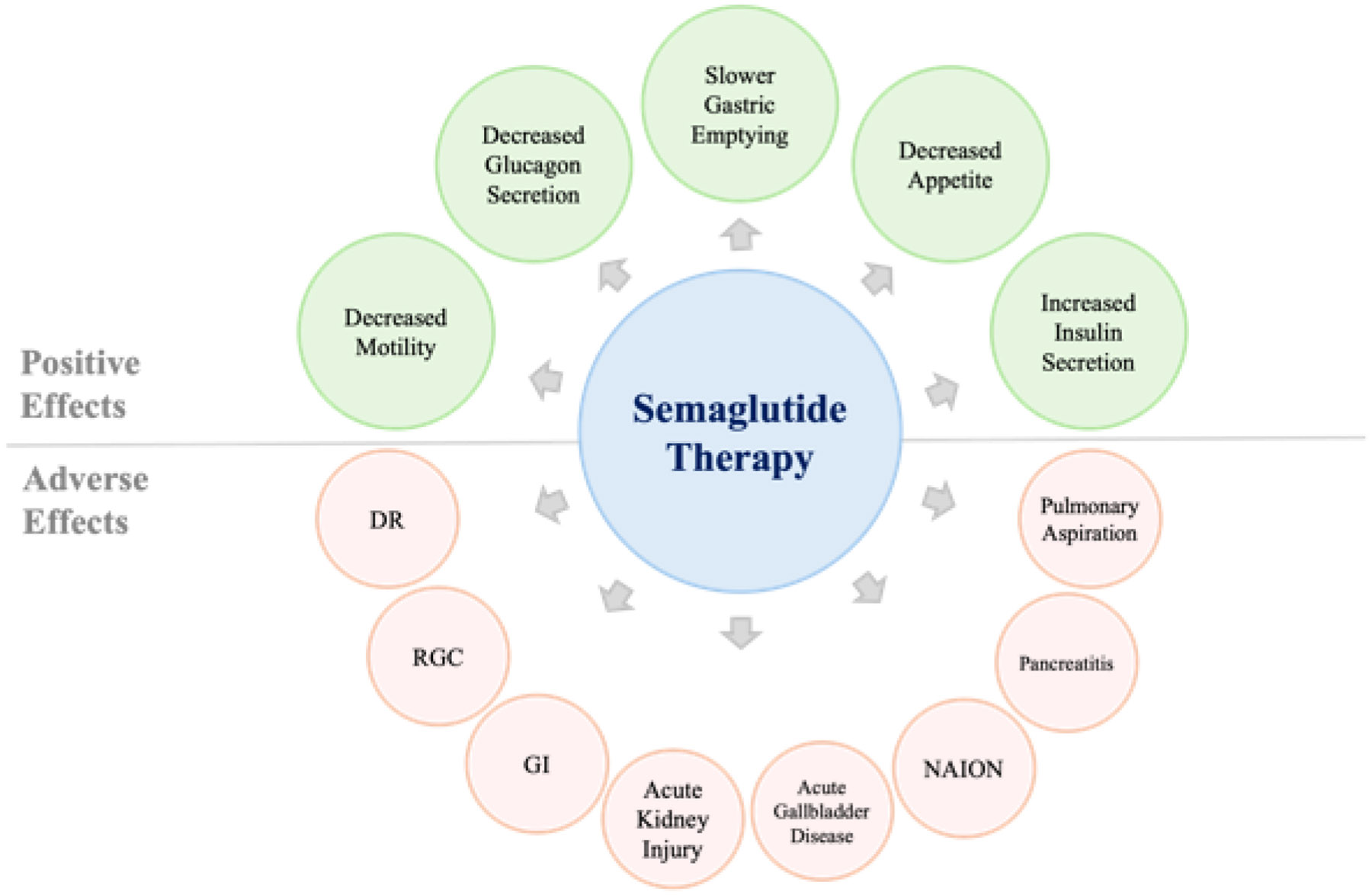
This figure provides a completed summary of positive effects and potential adverse effects, visually describing the diversity of adverse effects of semaglutide therapy.

**Figure 4:**
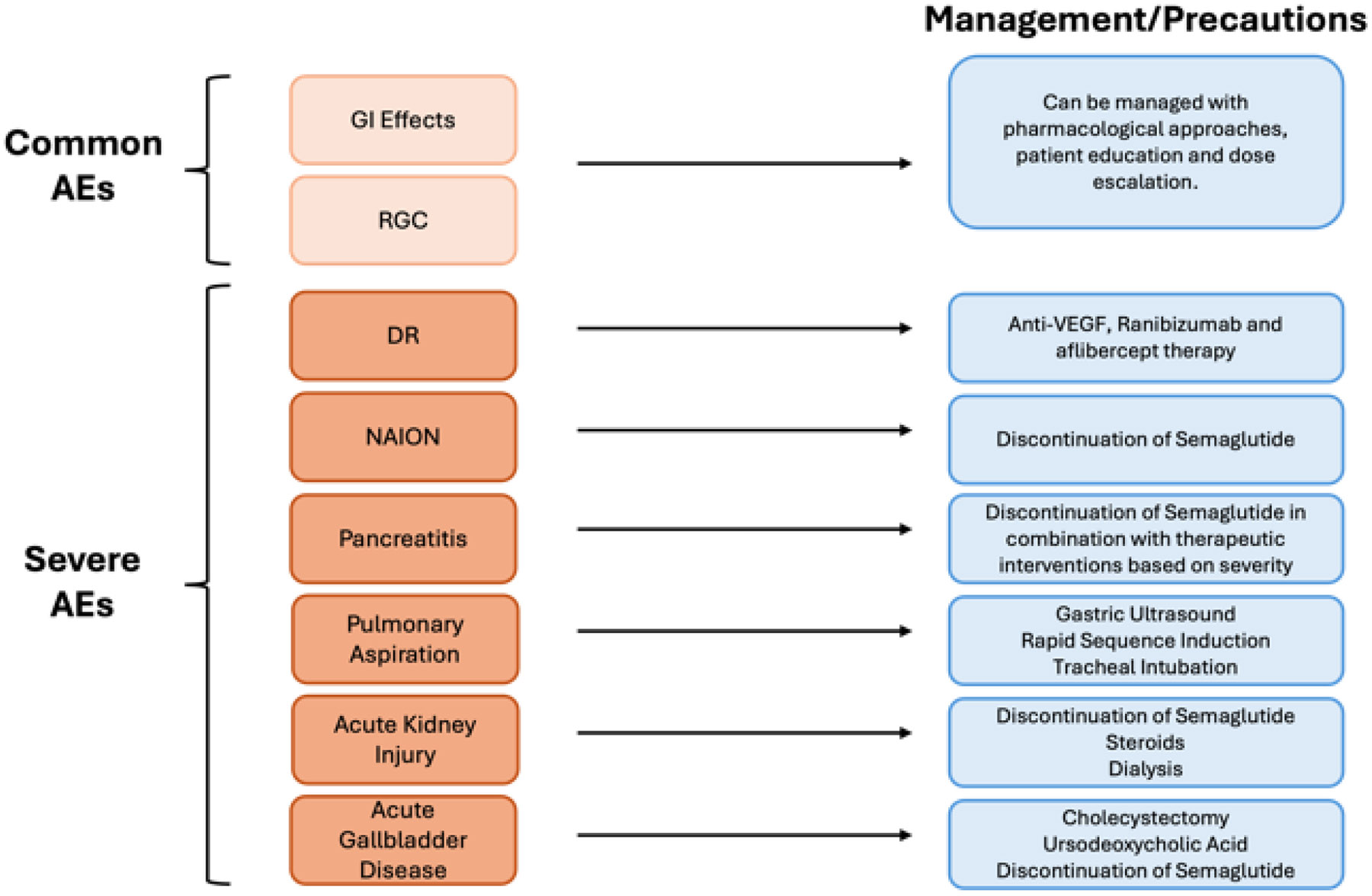
This figure depicts the common and severe adverse effects associated with semaglutide therapy along with methods to manage each. Common effects include gastrointestinal (GI) disturbances, like nausea and vomiting, and residual gastric content (RGC), which are generally mild and manageable. Severe and more rare effects include diabetic retinopathy (DR), pancreatitis, acute kidney injury, acute gallbladder disease, pulmonary aspiration, non-arteritic anterior ischemic optic neuropathy (NAION).

## IX. Key Findings

The key findings, outstanding questions and challenges identified in this study are:

- Semaglutide therapy can improve glycemic control, weight loss, and decrease cardiovascular risks like ischemic stroke.
- Semaglutide therapy is contraindicated in patient with medullary thyroid carcinoma and hence multiple endocrine neoplasia type 2.
- Semaglutide therapy is also contraindicated in pregnant patients due to case study evidence of unfavorable fetal outcomes, which the manufacturer has considered in drug packaging.
- Semaglutide can also be used to treat chronic kidney disease and polycystic ovary syndrome.
- Semaglutide therapy has adverse anesthetic risks due to delayed gastric emptying resulting in potential for residual gastric content and pulmonary aspiration when patients undergo general anesthesia.
- Other common adverse effects include gastrointestinal issues like nausea and vomiting while severe adverse effects include semaglutide induced pancreatitis, risk of diabetic retinopathy, acute gallbladder and kidney injury, etc.
- Semaglutide therapy can also delay absorption of drugs taken concurrently due to its ability to delay gastric emptying.
- Can semaglutide therapy be repurposed in Alzheimer’s Disease treatments due to its ability to regulate glucose transporters and cross the blood brain barrier, allowing it to prevent neurodegeneration?
- How should pre-operative guidelines be modified if a patient admits to semaglutide use which could result in anesthetic complications?
- How should patient dosages be standardized if they admit to simultaneous semaglutide therapy with other drug treatments considering the effect of delayed gastric emptying on absorption?
